# Factors associated with weight loss and health gains in a structured lifestyle modification programme for adults with severe obesity: a prospective cohort study

**DOI:** 10.3389/fendo.2023.1257061

**Published:** 2023-10-17

**Authors:** Francis M. Finucane, Irene Gibson, Robert Hughes, Enda Murphy, Lisa Hynes, Aisling Harris, Brian E. McGuire, Mary Hynes, Chris Collins, Kevin Cradock, Suzanne Seery, Jennifer Jones, Tim O’Brien, Martin J. O’Donnell

**Affiliations:** ^1^ Bariatric Medicine Service, Centre for Diabetes, Endocrinology and Metabolism, Galway University Hospitals, Galway, Ireland; ^2^ Department of Medicine, College of Medicine, Nursing and Health Sciences, University of Galway, Galway, Ireland; ^3^ Cúram, University of Galway, Galway, Ireland; ^4^ Croí, The West of Ireland Cardiac Foundation, Galway, Ireland; ^5^ National Institute of Preventive Cardiology, University of Galway, Galway, Ireland; ^6^ School of Psychology, University of Galway, Galway, Ireland; ^7^ Department of Upper Gastrointestinal Surgery, Galway University Hospital, Galway, Ireland; ^8^ Department of Health and Nutrition Sciences, Atlantic Technological University, Sligo, Ireland

**Keywords:** severe obesity, determinants of response, quality of life, anxiety, depression, diet, physical activity, structured lifestyle modification

## Abstract

**Background:**

Individual responses to behavioural weight loss interventions can vary significantly, and a better understanding of the factors associated with successful treatment might help to target interventions for those who will benefit the most. We sought to identify demographic and clinical characteristics that predicted intervention “success” (defined as ≥5% weight loss) and other health gains in patients with severe obesity attending a ten-week structured lifestyle modification programme.

**Methods:**

We conducted a prospective cohort study of all 1122 patients (751 (66.9%) female, mean age 47.3 ± 11.9 years, mean body mass index (BMI) 46.7 ± 7.8 kgm^-2^) referred from our hospital-based obesity clinic, who started the structured lifestyle programme between 2012-2019. We compared routine clinical measures such as weight, fitness, blood pressure, lipids and HbA1c at baseline and follow-up. We also used validated questionnaires to quantify anxiety, depression and health-related quality of life.

**Results:**

Of 1122 patients who started, 877 (78.2%) completed the programme and attended for follow up. Of these, 12.8% lost ≥5% body weight. The amount of weight lost was a strong and consistent predictor of improvements in metabolic, cardiovascular, and mental health, even after adjusting for age, sex, programme attendance and baseline fitness. Older age, male sex, being physically active and having lower anxiety and depression scores at baseline predicted greater weight loss. Younger age, depression and longer wait time to start the intervention were associated with drop-out.

**Conclusions:**

In adults with severe obesity completing a structured lifestyle modification programme, older age and good mental health were associated with programme completion and attaining ≥5% weight loss. The magnitude of weight lost was a strong predictor of improvements in cardiovascular, metabolic and mental health associated with programme completion.

## Background

Structured lifestyle modification programmes are an important part of the treatment of severe obesity (1), but drop-out rates can be high (2), weight loss is often modest (3), sustained reductions in body weight over time are difficult to maintain (4, 5) and adequate weight loss with associated health improvements (6, 7) may not be achieved. In a large general practice-based cohort of UK adults with severe obesity (typically defined as a body mass index (BMI) ≥40 kg m^-2^ (or ≥35 kg m^-2^ with co-morbidities such as type 2 diabetes or obstructive sleep apnoea syndrome)), the annual probability of achieving 5% weight loss was one in eight for men and one in seven for women (8). We have previously shown that patients with severe obesity who completed a ten-week, group-based, multidisciplinary structured diet and physical activity programme (CLANN - Changing Lifestyle with Activity and Nutrition) had reductions in body weight, cardiovascular risk factors, anxiety and depression scores and improvements in fitness and quality of life measures (9). However, there was significant variation in the magnitude of the response to the intervention among individuals.

This response heterogeneity is a common feature of behavioural interventions for obesity (10). A better understanding of the factors that contribute to this heterogeneity might allow for more effective and efficient delivery of “personalised” interventions (11), and the avoidance of ineffective strategies (12), for patients with obesity and related disorders. A comparison of the baseline characteristics of participants who remain in structured lifestyle modification programmes and those who “drop out” might allow the development of strategies to optimise participant retention and reduce attrition. This is particularly important in Ireland, where no previous studies have described the factors associated with participant retention in structured lifestyle programmes and where the widespread deployment of these interventions is imminent, as part of a new national clinical programme for the treatment of obesity (13).

Previous attempts to identify baseline characteristics that are good predictors of the magnitude of the response in individuals who complete behavioural interventions for obesity have had limited success. A recent systematic review and meta-analysis found that participants attending group-based rather than individual-level structured lifestyle modification programmes are 58% more likely to attain ≥5% weight loss (14). An Irish cohort study of participants who completed a 12-week structured diet and physical activity intervention for adults at high risk of diabetes found that standard clinical phenotypic characteristics were poor predictors of response to the intervention (15). In the Weight Loss Referrals for Adults in Primary Care (WRAP) trial in the UK, adults with overweight or obesity had no difference in weight loss after five years of follow-up according to ethnic group, gender, education level or household income, although older age was associated with greater weight loss (16). The BariDIET research group in the UK recently described how in adults with severe obesity undergoing a liver-reducing diet in preparation for bariatric surgery, older age, male sex and a diagnosis of diabetes were associated with a higher likelihood of attaining 5% weight loss (17). We sought to determine the association between weight loss and improvements in cardiovascular and metabolic health markers, in a ten-week structured lifestyle modification programme in patients living with severe obesity. We also sought to identify baseline anthropometric, demographic and clinical characteristics that were associated with programme completion or which predicted the amount of weight lost by programme completers.

## Methods

This single-centre, prospective cohort study was conducted according to STROBE guidelines (18). The overall changes in anthropometric and metabolic characteristics in participants who completed the programme have been described in detail previously (9, 19). The study population consisted of patients referred from our hospital-based bariatric outpatient clinic to our community-based structured lifestyle modification programme between 2012 and 2019. Inclusion criteria for the intervention included being over 18 years old and having a body mass index (BMI) ≥40 kgm^-2^ (or ≥35 kgm^-2^ with obesity-related comorbidities). Patients with cognitive impairment, poorly controlled hypertension (>180/110 mmHg) (20), symptoms suggestive of cardiac ischaemia or those who could not walk 10 metres without assistance were excluded from the intervention. For these analyses, we excluded patients who were on obesity medications, or who had missing baseline or follow-up weight measures.

During the first programme visit, participants had demographic, anthropometric and clinical data recorded including medical history and relevant medication usage (statin, antihypertensive and antiplatelet drugs). Weight was measured on a Seca^®^ 877 scale and height was measured using a Seca^®^ Leicester wall-mounted stadiometer. Blood pressure was measured from the right arm, using an Omron^®^ 705IT oscillometric device. Self-reported physical activity was quantified using seven-day activity recall, in order to determine whether participants were achieving targets for moderate intensity aerobic activity of 150 minutes per week (21). The Incremental Shuttle Walk Test (ISWT) was used to derive an estimate of maximal “Metabolic Equivalent of Task” (Est MET_max_) (22). The Hospital Anxiety and Depression Scale (HADS) was used to obtain self-reported anxiety and depression scores (23), with scores >11 (from a total possible score of 21) being considered “high”. Self-reported quality of life was measured using the European Quality of Life Questionnaire Visual Analogue Scale (EQVAS) (24), with a possible score from 0 (worst) to 100 (best), and the Dartmouth COOP Questionnaire (25), which included nine domains with possible scores ranging from 1 (best) to 5 (worst), listed in **Table 1**.

**Table 1:** Baseline anthropometric and metabolic characteristics of patients with severe obesity who completed or did not complete the CLANN structured lifestyle modification programme.

Variable Name	Completers			Non-Completers			p-value
n	877			245			
Age (years)	48	[40,	56]	45	[37,	53]	**0.0015**
Sex:							
Female, n	578	(65.9%)		173	(70.6%)		0.17
Current smoker, n	107	(12.3%)		39	(16.4%)		0.095
Attaining Activity Target, n	46	(5.6%)		16	(7%)		0.42
Comorbidities:							
Depression, n	167	(28.6%)		67	(41.1%)		**0.002**
Diabetes, n	230	(26.3%)		57	(23.5%)		0.37
CVD, n	16	(1.8%)		3	(1.2%)		0.52
Sleep Apnoea, n	108	(19.5%)		27	(17.8%)		0.63
Osteoporosis, n	17	(2%)		4	(1.7%)		0.77
Arthritis, n	304	(35.4%)		88	(36.4%)		0.78
Chronic Back Pain, n	410	(47.3%)		118	(48.8%)		0.69
Dyslipidaemia, n	445	(53.7%)		114	(57%)		0.41
Hypertension, n	326	(37.2%)		94	(38.7%)		0.68
ESC Risk Score	0	[0,	0]	0	[0,	0]	0.97
ESC Risk Status:							
Low, n	371	(75.6%)		96	(76.2%)		0.68
Medium, n	106	(21.6%)		27	(21.4%)		
High, n	10	(2%)		1	(0.8%)		
Very High, n	4	(0.8%)		2	(1.6%)		
Medications:							
Antiplatelet, n	147	(16.8%)		29	(11.8%)		0.061
Betablocker, n	122	(13.9%)		28	(11.4%)		0.31
ARB, n	172	(19.6%)		35	(14.3%)		0.057
ACEi, n	135	(15.4%)		27	(11%)		0.085
Statin, n	232	(26.5%)		51	(20.8%)		0.072
Obesity Medication, n	14	(1.6%)		1	(0.4%)		0.15
Diabetes Medication, n	243	(27.7%)		57	(23.3%)		0.17
Anthropometric:							
Weight (Kg)	128.1	[114.1,	144.4]	126	[110,	142.2]	**0.033**
BMI (Kg m^-2^)	46	[41.5,	51.7]	44.9	[40.8,	51]	0.081
Excess Weight (%)	84	[66,	106.8]	79.8	[63.2,	104]	0.081
Waist (cm)	137	[126,	149.5]	134	[123.5,	147.1]	**0.019**
Systolic BP (mmHg)	129	[119.8,	140]	127	[118,	137.5]	0.058
Diastolic BP (mmHg)	85	[78,	91.5]	85.5	[78.5,	92.5]	0.49
Maximum METs	4.6	[4.3,	5.8]	4.6	[4.3,	6.4]	0.99
Metabolic:							
Total Cholesterol (mmol/l)	4.6	[4,	5.4]	4.7	[4.2,	5.4]	0.095
LDL Cholesterol (mmol/l)	2.8	[2.1,	3.4]	2.9	[2.3,	3.4]	0.082
HDL Cholesterol (mmol/l)	1.2	[1,	1.4]	1.1	[1,	1.3]	0.68
Triglycerides (mmol/l)	1.5	[1.1,	2]	1.5	[1.1,	2.2]	0.35
HbA1c (mmol/mol) (No DM)	37	[34,	40]	36	[34,	39]	0.35
HbA1c (mmol/mol) (DM)	53	[43,	65]	47.5	[44,	66]	0.56
HADS Scores:							
HADS Anxiety Score >11, n	263	(31.2%)		81	(37%)		0.1
HADS Depression Score >11, n	190	(22.5%)		67	(30.6%)		**0.013**
Anxiety Score	8	[5,	12]	9	[6,	12]	**0.052**
Depression Score	7	[4,	10]	8	[5,	12]	**0.041**
EQVAS Score	50	[35,	70]	50	[35,	66.5]	0.29
Dartmouth COOP:							
Dartmouth Physical	4	[3,	4]	4	[3,	5]	1
Dartmouth Feelings*	3	[2,	4]	3	[2,	4]	**0.021**
Dartmouth Daily Activity	3	[2,	4]	3	[2,	4]	0.39
Dartmouth Social Activity	2	[1,	4]	2	[1,	4]	0.4
Dartmouth Pain	3	[2,	4]	3.5	[2,	4]	0.2
Dartmouth Change	3	[3,	3]	3	[3,	3]	0.23
Dartmouth Overall Health*	4	[3,	4]	4	[3,	4]	**0.034**
Dartmouth Support	2	[1,	3]	2	[1,	4]	0.087
Dartmouth Quality of Life	3	[2,	3]	3	[2,	3]	0.17

Continuous variables are presented as medians and [inter-quartile range], as none were normally distributed.

Proportions are expressed as the number of participants, n, and (percentage).

Comparisons of baseline measures of continuous variables between programme completers and non-completers were made using the Wilcoxon Rank Sum (Mann-Whitney U) Test. Comparisons of proportions between the two groups for categorical variabels were made using the Chi-Square test.

*In completers and non-completers, the mean (± standard deviation) Dartmouth Feelings scores were 2.7 ± 1.2 versus 2.9 ± 1.3, respectively and the mean (± standard deviation) Dartmouth Overall Health scores were 3.6 ± 1.2 versus 3.7 ± 1, respectively.

ACEi, Angiotensin Converting Enzyme inhibitor.

ARB, Angiotensin Receptor Blocker.

BP, Blood Pressure.

BMI, Body Mass Index.

EQVAS, European Quality of Life Questionnaire Visual Analogue Scale.

ESC, European Society of Cardiology.

HADS, Hospital Anxiety and Depression Scale.

HDL, High Density Lipoprotein.

LDL, Low Density Lipoprotein.

MET, Metabolic Equivalent of Task. P values <0.05 have been highlighted in bold.

All blood samples were analysed locally in the Galway University Hospitals’ Department of Clinical Biochemistry (certified to ISO 15189 2007 accreditation standard). HbA1c was measured with HPLC (Menarini^®^ HA8160 auto-analyser). Total cholesterol was measured using the CHOD-PAP method. High density lipoprotein (HDL)-cholesterol and triglycerides were measured using the enzymatic and the GPO-PAP methods, respectively (Roche COBAS^®^ 8000 modular analyser). Low density lipoprotein (LDL)-cholesterol was derived with the Friedewald equation (26).

At the first programme visit, for each patient, an individualised exercise prescription and risk stratification took place in order to ensure that they had adequate progression of exercise intensity over the duration of the programme. Thereafter, weekly group-based sessions lasting 2.5 hours each took place over eight consecutive weeks. These consisted of an educational workshop combined with a physical activity class. Exercise was performed without any specialist equipment in order to encourage continuation of the activity beyond the duration of the programme. Specific attention was given to reducing sedentary behaviour. For each participant, a dietary assessment was completed by the dietitian to assess eating patterns, nutritional adequacy and nutrition-related knowledge and skills. Dietary advice to participants was based on the European guidelines for cardiovascular prevention (27). A target of weekly weight loss of 0.5kg was encouraged through a cardioprotective diet with an energy deficit of 600kcal/day. For the current analysis, we defined ‘treatment success’ as loss of 5% or more of baseline body weight (28).

The educational component consisted of workshops specific to diet (healthy eating principles, portion control, food labelling), exercise, physical activity, cardiovascular health, stress management and psychological issues relevant to people with obesity. Established individual and group-based motivational interviewing strategies were used throughout the programme, in order to enhance self-efficacy in achieving goals (29). Blood pressure and lipid targets were based initially on the 2012 European Society of Cardiology (ESC) prevention guidelines (27) when the blood pressure target was 140/90 mmHg (140/85 mmHg in patients with type 2 diabetes) and the lipid targets were total cholesterol <5 mmol/l, LDL cholesterol <3 mmol/l and triglycerides <1.7 mmol/l. When the guidelines were updated in 2016 (30), we sought an LDL cholesterol <1.8 mmol/l in patients with established cardiovascular disease, and again in 2018 we revised our blood pressure target downwards in patients with diabetes to <130/80 mmHg (20). At the last programme visit at 10 weeks, all baseline measures were repeated. The study was approved by the Galway University Hospitals Research Ethics Committee (reference CA 1070). All participants provided written informed consent for their data to be used in these analyses.

### Statistical analyses

As this was a convenience sample of patients referred from our bariatric clinic to a structured lifestyle modification programme, we did not conduct an *a priori* power analysis to determine the sample size. Summary statistics are presented as numbers and percentages for categorical variables, means ± standard deviations for normally distributed continuous variables and for variables that were not normally distributed, medians and [inter-quartile range]. In order to identify potential baseline predictors of intervention “success” in programme completers, (i.e., weight loss above 5%) we used univariate and stepwise backward multivariate logistic regression, with a threshold of *p*>0.1 for hierarchical removal of predictor variables. The potential predictor variables we included were age, sex, waiting time to start the intervention, travel distance, baseline fitness and BMI, smoking, employment status, GMS eligibility, living with partner, pre-existing diabetes, depression, arthritis, back pain, dyslipidaemia, cardiovascular disease, hypertension, the presence of high anxiety and depression scores (>11) and medication usage. We set a threshold of p>0.1 for hierarchical removal of predictor variables.

We also examined associations between baseline predictor “exposure” variables and weight loss as a continuous “outcome” dependent variable, using linear regression. Then, associations between the magnitude of weight loss percentage (as the independent/exposure variable) and changes in cardiovascular, metabolic, psychological and quality of life (dependent/outcome variables) were also quantified using linear regression. Comparisons of these variables according to weight loss category (no weight loss/weight gain, 0-4.99% weight loss or ≥5% weight loss) were made using one way Analysis of Variance (ANOVA) or the Kruskal Wallis test. Stata^®^ version 17 was used for all analyses. Comparisons of baseline characteristics in programme completers versus non-completers were made using the Wilcoxon Rank Sum Test or the Chi-Square test for continuous and categorical variables, respectively. Identification of baseline characteristics that predicted programme completion was undertaken using univariate and stepwise backward multivariate logistic regression analysis. We set a threshold of p>0.1 for hierarchical removal of predictor variables.

## Results

**Figure 1:**
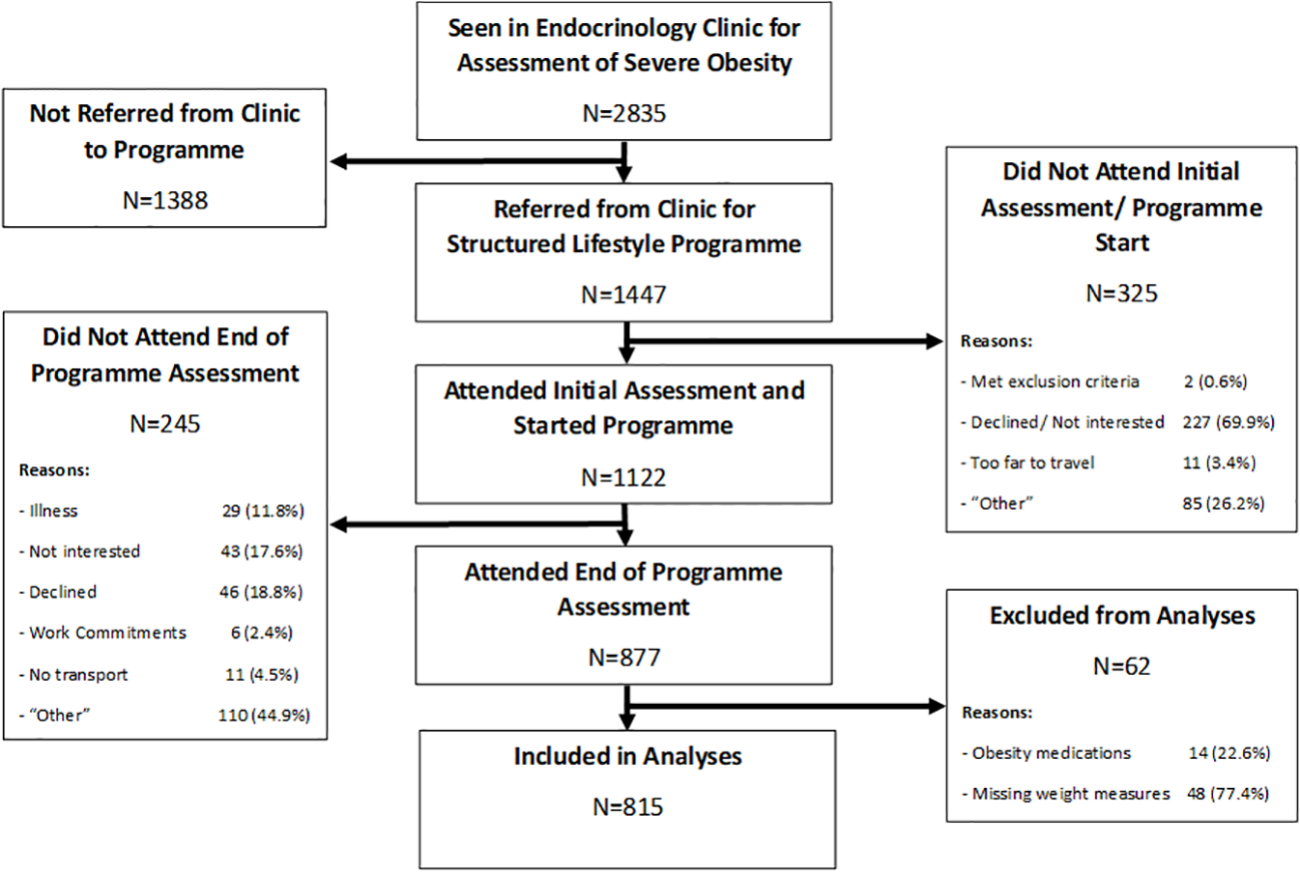
Flow Diagram of Participant Recruitment.

Between 2012 and 2019, 2,835 patients with severe obesity were seen in our hospital-based bariatric outpatient clinic, as shown in **Figure 1**. Of these, 1,447 (51%) were referred to the CLANN structured lifestyle modification programme, 1,122 (77.5%) of whom attended for the initial programme assessment. In total, 877 (78.2%) of those participants completed the programme and attended the follow-up assessment visit, while 245 (21.8%) dropped out prior to completion. For the current analyses, 62 patients were excluded either because of concurrent obesity medication usage, or because baseline or follow-up weight measures were missing, with 815 programme completers included in analyses. The baseline anthropometric, metabolic and clinical characteristics of all 1122 programme starters are shown in **Table 1**, divided according to whether they completed the programme or not. (The equivalent baseline demographic characteristics are shown in **Supplementary Table 1**).

### Baseline characteristics associated with lifestyle intervention completion

Compared to participants who dropped out of the programme, those who completed the programme were statistically significantly older, with a higher body weight and waist circumference, and were less likely to have a pre-existing diagnosis of depression, score above 11 units for depression on the HADS scale and they had a lower median depression score. Completers also scored better in two domains on the Dartmouth COOP questionnaire – “feelings” and “overall health”. Although there was a lower proportion of participants who were entitled to means-tested, state-sponsored medical care (“General Medical Services”, GMS) in the group completing the intervention compared to non-completers, there were no differences in the proportions of completers by employment-, education- or marital status. There was no difference in in the distance travelled from home to the intervention in completers versus non-completers, nor did the proportions of those who had comorbidities other than depression, took various medications, had higher cardiovascular risk or smoked differ between completers and non-completers.

In univariate logistic regression analyses, for every year older and for every unit increase in BMI, there was a 2% higher likelihood of programme completion, as shown in **Table 2**. Conversely, participants with a GMS entitlement, those on disability state benefit (“sick” employment status), those with a pre-existing diagnosis of depression or those with a high depression score were less likely to complete the programme, as shown. In stepwise backward hierarchical multiple logistic regression, with programme completion as the dependent categorical variable, we found that for every 10 days participants had to wait to start the programme, the likelihood of dropout increased by 2% and that higher BMI was associated with a greater likelihood of programme completion, as shown in **Table 2**. Participants with a pre-existing diagnosis of depression were 54% less likely [95% confidence interval 24 to 73%] to complete the programme, as shown. Results of these multivariate analyses were the same when *p* value thresholds of >0.05 or >0.2 were used for hierarchical removal of predictor variables.

**Table 2:** Logistic regression analyses of potential baseline variables for prediction of programme completion in participants who started the programme.

	Univariate Logistic Regression			Multivariate Logistic Regression		
	Odds Ratio	95% Confidence Interval	p-value	Odds Ratio	95% Confidence Interval	p-value
Age (years):	1.02	[1.01, 1.03]	**0.004**	1.01	[0.98, 1.04]	0.47
Male sex:	1.24	[0.91, 1.69]	0.17	1.23	[0.71, 2.14]	0.47
Wait time to start programme (days):	0.999	[0.998, 1.000]	0.051	0.998	[0.996, 1.000]	**0.02**
Distance to centre (Km):	1.00	[1.00, 1.00]	0.84	1.00	[1.00, 1.01]	0.59
Baseline BMI (kg m^-2^):	1.02	[1.00, 1.04]	**0.045**	1.04	[1.01, 1.07]	**0.023**
Smoking Status	0.71	[0.48, 1.06]	0.096	1.83	[0.81, 4.12]	0.15
Employment Category						
- Self employed	0.78	[0.42, 1.45]	0.44	0.62	[0.25, 1.53]	0.30
- Unemployed	1.01	[0.61, 1.68]	0.96	1.58	[0.67, 3.76]	0.30
- Retired	1.31	[0.75, 2.31]	0.34	1.89	[0.68, 5.28]	0.22
- Looking after family	0.87	[0.57, 1.34]	0.54	1.11	[0.54, 2.28]	0.77
- Student/training scheme	0.81	[0.40, 1.62]	0.55	3.47	[0.70, 17.30]	0.13
- Sick	0.54	[0.34, 0.85]	**0.008**	0.81	[0.38, 1.74]	0.60
Lives with partner	1.08	[0.80, 1.44]	0.62	1.25	[0.75, 2.07]	0.39
GMS eligible	0.67	[0.47, 0.94]	**0.02**	0.69	[0.38, 1.24]	0.21
Achieving Physical Activity Targets	0.78	[0.44, 1.41]	0.42	0.76	[0.28, 2.04]	0.58
Type 2 Diabetes	1.17	[0.84, 1.63]	0.37	0.76	[0.43, 1.34]	0.35
Hypertension	1.36	[0.95, 1.95]	0.09	1.00	[0.59, 1.70]	0.99
Cardiovascular Disease	1.49	[0.43, 5.17]	0.53	2.78	[0.32, 23.85]	0.35
Arthritis	0.96	[0.71, 1.29]	0.78	1.05	[0.61, 1.79]	0.87
Back pain	0.94	[0.71, 1.25]	0.69	1.05	[0.65, 1.71]	0.83
Dyslipidaemia	0.88	[0.64, 1.20]	0.41	0.91	[0.56, 1.49]	0.70
Depression	0.58	[0.40, 0.82]	**0.003**	0.46	[0.27, 0.76]	**0.003**
High Depression Score (≥11)	0.66	[0.47, 0.92]	**0.014**	0.71	[0.39, 1.30]	0.27
High Anxiety Score (≥11)	0.77	[0.57, 1.05]	0.10	1.37	[0.78, 2.41]	0.27
Medication usage						
- - Antiplatelet therapy (%)	1.50	[0.98, 2.30]	0.062			
- - Betablocker (%)	1.25	[0.81, 1.94]	0.31			
- - ACEi (%)	1.47	[0.95, 2.28]	0.087			
- - ARB (%)	1.46	[0.99, 2.17]	0.058			
- - Statin (%)	1.37	[0.97, 1.93]	0.073			
- - Diabetes Medication	1.26	[0.91, 1.76]	0.17			

ACEi, Angiotensin Converting Enzyme inhibitor.

ARB, Angiotensin Receptor Blocker.

BMI, Body Mass Index.

GMS, General Medical Services.

The multivariate logistic regression models used a stepwise, backward hierarchical approach to determine odds ratios and [95% confidence intervals] for achieving 5% or more weight loss, according to baseline predictor variables, with a p-value threshold for removal from the model of >0.1. In multivariate models, all medication use catgories were removed. P values <0.05 have been highlighted in bold.

### Baseline characteristics predicting good response to lifestyle intervention

**Figure 2:**
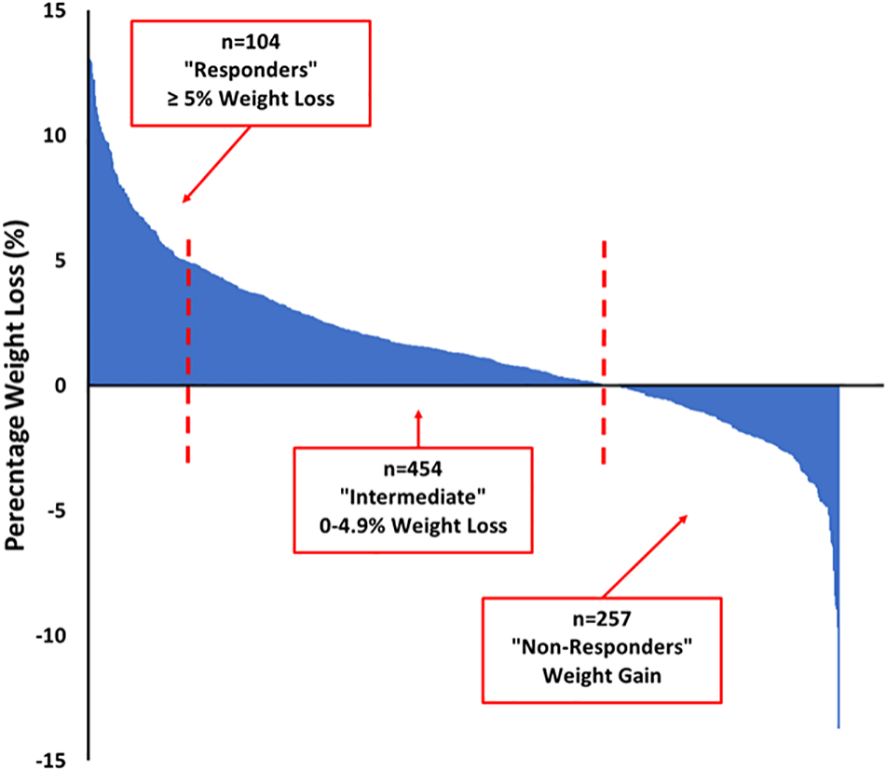
Waterfall Plot of Percentage Weight Loss in CLANN Programme Completers.

Of the 877 participants who completed the programme and attended for follow-up measures, 14 were taking obesity medications (liraglutide or orlistat) and were excluded from the longitudinal cohort analysis. Baseline or follow-up weight was missing in 48 of the remaining 863 participants. In the 815 participants for whom baseline and follow-up weight measures were available, the median weight loss was 1.6 [-0.5, 4.4] kg (range -18.2 to 20.4 kg) and the median weight loss percent was 1.3 [-0.4, 3.3] % (range -13.7 to 15.4%). As shown in the Waterfall plot in **Figure 2**, there was no weight loss or some weight gain in 257 (31.5%) participants (“non-responders”), 454 (55.7%) lost between 0.1 and 4.99% (“intermediate responders”) and 104 (12.8%) of participants lost 5% or more of their total body weight (“responders”). As shown in **Table 3**, responders to the intervention were older, more likely to be male and to report meeting physical activity targets at follow-up. They attended more of the weekly programme sessions. Responders were less likely to have depression or to score highly on HADS anxiety or depression scores. Responders also scored better on several domains of the Dartmouth COOP questionnaire. Responders had lower total cholesterol and triglyceride levels, but slightly higher HbA1c values (in those without diabetes) compared to non-responders.

**Table 3:** Baseline participant characteristics according to category of weight loss during the CLANN structured lifestyle modification programme.

Variable Name	All Participants	Non-Responders (No weight loss/weight gain)	Intermediate Responders (0.1-4.99% Weight loss)	Responders (≥5% weight loss)	p-value
N	815	257	454	104	
Age (Years):	48 ± 11.7	46.6 ± 12	48.4 ± 11.8	50 ± 10.4	0.037*
Wait Time (Days)	74.5 [28, 172]	69 [29, 175]	78 [27, 173.5]	81 [28, 159]	0.95
Distance >40 km (%)	66.1	66.4	65.4	68.3	0.85
Distance (Km)	65 [25, 100]	60 [25, 100]	60 [25, 97]	72.5 [25, 105]	0.43
Female (%)	66.4	71.6	66.7	51.9	**0.002**
Programme Attendance:					
Attended <6 sessions (%)	25.4	28.8	24.9	19.2	0.16
Attended ≥6 sessions (%)	74.6	71.2	75.1	80.8	
Sessions Attended (n):	7 [5, 7]	6 [5, 7]	7 [6, 8]	7 [6, 8]	**0.0001**
Achieved PA Target:					
At End of Programme (%)	37	26.7	40.4	47.9	**<0.0001**
At Start of Programme (%)	5.6	3.6	5.9	9.5	0.095
Marital Status:					
Divorced/Separated (%)	7.9	6.4	8.3	9.6	0.19
Married (%)	53.7	54	52.5	58.7	
Permanent Partnership (%)	6	8.3	4.3	7.7	
Single (%)	29.9	28.2	32.5	23.1	
Widowed (%)	2.5	3.2	2.5	1	
Lives with partner (%):	61.3	62	59.4	67.7	0.29
Education:					
Primary or less (%)	27.7	28.8	26.1	31.5	0.79
Secondary/Vocational (%)	34.8	33.2	36.4	31.5	
College/University/PG (%)	37.6	38	37.4	37.1	
GMS Eligible (%):	67.7	69.1	68.5	61.1	0.33
Employment Status:					
Employed (%)	35.1	34.2	34.5	40	0.64
Self-employed (%)	6.5	8.1	5.2	8	
Unemployed (%)	13.1	11.4	14.3	12	
Retired (%)	12.2	11	13.6	9	
Caring for family (%)	17.7	17.5	18.6	14	
Student (%)	5	6.5	4.1	5	
Sick (%)	10.6	11.4	9.8	12	
Current Smoker (%):	11.6	13.7	10.9	9.6	0.43
Drinks Alcohol (%):	38.7	35.7	39.8	42.4	0.52
CVD Risk Category:					
ESC Risk Group Low (%)	75	75.7	75.9	69	0.69
ESC Risk Group Medium (%)	22.2	20.3	21.7	29.3	
ESC Risk Group High (%)	2.2	2.7	2	1.7	
ESC Risk Group Very High (%)	0.7	1.4	0.4	0	
Comorbidities:					
Diabetes (%)	26	26.2	26.5	23.1	0.77
Prevalent CVD (%)	5.3	3.5	6.4	4.8	0.25
Sleep Apnoea (%)	19.1	15.3	20.7	23	0.26
Arthritis (%)	35.5	39.1	34	33.7	0.37
Hypertension (%)	47.5	46.5	47.7	49.2	0.93
Chronic Back Problems (%)	47.3	51.2	45.9	44.1	0.31
Osteoporosis (%)	2.1	2	2.2	1.9	0.96
Depression (%)	28	28.3	30.6	15.6	**0.055**
Antiplatelet therapy (%)	16.6	17.5	17	12.5	0.48
Betablocker (%)	13.4	12.1	13.9	14.4	0.75
ACEi (%)	15.3	11.7	16.3	20.2	0.088
ARB (%)	19.5	21.8	18.1	20.2	0.48
Statin (%)	26.5	24.9	28.9	20.2	0.15
Anthropometric:					
Weight (Kg)	128.5 [114.2, 144.4]	128.9 [114.1, 142.4]	128.2 [114.4, 144.4]	128.1 [113, 154.1]	0.8
BMI (Kg m^-2^)	46 [41.7, 51.8]	46.1 [42, 52]	46.1 [41.9, 51.6]	45.5 [40.6, 52.1]	0.51
Excess Weight (%)	84.1 [67, 107.1]	84.4 [68.1, 108]	84.2 [67.7, 106.6]	81.8 [62.3, 108.2]	0.51
Waist (cm)	137 [126, 149.4]	137.2 [126, 149]	137 [127, 150]	137 [126, 149]	0.98
Systolic BP (mmHg)	129 [120, 140]	129 [118, 140]	129 [121, 140]	130 [122.8, 141.3]	0.51
Diastolic BP (mmHg)	85 [78.5, 91.5]	85 [79, 91.5]	86 [79, 91.5]	83.5 [76, 92]	0.29
Maximum METs	4.6 [4.3, 5.7]	4.6 [4.3, 5.4]	4.6 [4.3, 5.7]	4.8 [4.3, 6.9]	0.83
Metabolic:					
Total Cholesterol (mmol/l)	4.6 [4, 5.4]	4.8 [4.1, 5.5]	4.5 [3.9, 5.3]	4.7 [3.9, 5.3]	0.049*
LDL Cholesterol (mmol/l)	2.8 [2.1, 3.4]	2.9 [2.3, 3.6]	2.7 [2.1, 3.3]	2.9 [2.1, 3.5]	0.19*
HDL Cholesterol (mmol/l)	1.2 [1, 1.4]	1.2 [1, 1.4]	1.1 [1, 1.4]	1.1 [0.9, 1.3]	0.019*
Triglycerides (mmol/l)	1.5 [1, 2]	1.5 [1.1, 2]	1.5 [1.1, 2]	1.3 [1, 1.8]	0.021*
HbA1c (mmol/mol) (No DM)	37 [34, 40]	35 [33, 39]	37 [34, 41]	37 [36, 40]	**0.0085**
HbA1c (mmol/mol) (DM)	53 [42, 66]	51 [40.5, 71]	54 [44, 65]	53 [43, 67]	0.75
HADS Scores:					
Depression >11 (%)	21.8	25.9	21.3	14.1	**0.052**
Anxiety >11 (%)	31	35.2	31	20.2	**0.024**
Depression Score	7 [4, 10]	7 [4, 11]	7 [4, 10]	7 [3, 13]	0.32
Anxiety Score	8 [5, 12]	9 [5, 12]	8 [5, 12]	9 [5, 12]	0.0087*
EQVAS Score	50 [35, 70]	50 [30, 70]	50 [40, 70]	50 [36, 70]	0.24
Dartmouth COOP:					
Dartmouth Physical	4 [3, 5]	4 [3, 5]	4 [3, 5]	3 [3, 4]	0.045*
Dartmouth Feelings	3 [2, 4]	3 [2, 4]	3 [2, 4]	2 [1, 3.5]	0.022*
Dartmouth Daily Activity	3 [2, 4]	3 [2, 4]	3 [2, 3]	2.5 [2, 3]	0.039*
Dartmouth Social Activity	2 [1, 4]	2 [1, 4]	2 [1, 4]	2 [1, 3]	0.17*
Dartmouth Pain	3 [2, 4]	3 [2, 4]	3 [2, 4]	3 [2, 4]	0.1*
Dartmouth Change	3 [3, 3]	3 [3, 3]	3 [3, 3]	3 [3, 3]	0.23*
Dartmouth Overall Health	4 [3, 4]	4 [3, 4]	4 [3, 4]	3 [3, 4]	0.0015*
Dartmouth Support	2 [1, 3]	2 [1, 3]	2 [1, 3]	2 [1, 3]	0.2*
Dartmouth Quality of Life	3 [2, 3]	3 [2, 3]	3 [2, 3]	2.5 [2, 3]	0.07*

“Non-responders” were defined as having no weight loss or some weight gain, “intermediate responders” lost between 0.1 and 4.99% and “responders” were defined as those who lost 5% or more of their total body weight.

Proportions of categorical variables are presented as percentages, while continuous variables are presented as medians and interquartile ranges, as they are not normally distributed. Comparisons of proportions of independent/predictor categorical variables across the three weight loss categories have been made with the Chi-Square test. Comparisons of normally distributed independent/exposure variables have been made with one-way analysis of variance and the Bonferroni (pairwise) test. Comparisons of median independent/exposure variables have been made with the Kruskal Wallis test and the Dunn (pairwise) test.

*Denotes p value for pairwise comparison between non-responders and responders.

ACEi, Angiotensin Converting Enzyme inhibitor.

ARB, Angiotensin Receptor Blocker.

GMS, General Medical Services, refers to the means-tested provision of state-sponsored care.

HADS, Hospital Anxiety and Depression Scale.

KW, Kruskal Wallis.

PA, Physical Activity. P values <0.05 have been highlighted in bold.

In order to identify baseline characteristics which could reliably predict weight loss success, we used stepwise backward hierarchical multiple logistic regression, with the attainment of ≥5% weight loss as the dependent categorical variable, as shown in **Table 4**. In the multivariate model, males were 3.6 [1.69, 7.73] times more likely to achieve 5% weight loss or more than females (*p*=0.001). Retired participants were 95% [51, 99] less likely (*p*=0.01) than non-retired participants, and those with a high anxiety score (>11 units) were 75% [30, 91] less likely (*p*=0.009) than those without a high anxiety score, to achieve 5% weight loss. However, age, waiting time to start the programme, baseline fitness and baseline BMI were not associated with the likelihood of achieving ≥5% weight loss, as shown in **Table 4**. Those travelling further to participate in the intervention were 1% more likely to have weight loss success for every kilometre they had to travel. Results were similar when a *p* value threshold of >0.2 was used for removal of candidate predictor variables, and when a non-hierarchical stepwise approach was used (with a *p* value threshold for inclusion of 0.05).

**Table 4:** Multivariate logistic regression analyses of potential baseline variables for prediction of weight loss ≥5% in programme completers.

	Multivariate Logistic Regression		
	Odds Ratio	95% Confidence Interval	p-value
Age (years):	1.03	[0.98, 1.07]	0.28
Male sex:	3.61	[1.69, 7.73]	**0.001**
Wait time to start programme (days):	1.00	[1.00, 1.00]	0.98
Distance to centre (Km):	1.01	[1.00, 1.01]	**0.042**
Baseline fitness (METs):	1.02	[0.84, 1.24]	0.82
Baseline BMI (kg m^-2^):	1.01	[0.96, 1.06]	0.69
Smoking Status	0.97	[0.29, 3.31]	0.96
Employment Category			
- Self employed	0.24	[0.05, 1.24]	0.088
- Unemployed	1.42	[0.50, 4.05]	0.51
- Retired	0.05	[0.01, 0.49]	**0.010**
- Looking after family	0.66	[0.21, 2.09]	0.48
- Student/training scheme	1.44	[0.23, 9.10]	0.48
- Sick	0.47	[0.12, 1.87]	0.29
Lives with partner	1.98	[0.90, 4.39]	0.090
GMS eligible	1.46	[0.65, 3.26]	0.36
Achieving Physical Activity Targets	1.46	[0.35, 6.17]	0.61
Type 2 Diabetes	0.83	[0.34, 2.02]	0.68
Hypertension	1.13	[0.53, 2.44]	0.75
Cardiovascular Disease	2.43	[0.21, 28.80]	0.48
Arthritis	0.66	[0.30, 1.46]	0.30
Back pain	1.41	[0.69, 2.88]	0.35
Dyslipidaemia	0.90	[0.44, 1.83]	0.77
Depression	0.48	[0.17, 1.33]	0.16
High Depression Score (≥11)	0.85	[0.29, 2.50]	0.77
High Anxiety Score (≥11)	0.25	[0.09, 0.70]	**0.009**
Antiplatelet therapy (%)	0.28	[0.07, 1.14]	0.077

BMI, Body Mass Index.

GMS, General Medical Services.

MET, Metabolic Equivalent of Task.

The multivariate logistic regression models used a stepwise, backward hierarchical approach to determine odds ratios and [95% confidence intervals] for achieving 5% or more weight loss, according to baseline predictor variables, with a p-value threshold for removal from the model of >0.1.

Apart from antiplatelet therapy, all other medications were removed from the multivariate model. P values <0.05 have been highlighted in bold.

### Changes in anthropometric-, metabolic- and mental health-related outcomes according to weight loss category

Differences in the magnitude of the changes in various outcomes across the three weight loss categories are shown in **Table 5**. There was a greater reduction in diastolic blood pressure in participants who lost ≥5% of their body weight compared to intermediate- or non-responders (p=0.029), but not in systolic blood pressure. The estimated MET_max_ from the incremental shuttle walk test increased by 1 MET in responders compared to 0.7 METs in intermediate or non-responders (p=0.0025). Similarly, there were greater reductions in total-, HDL- and LDL-cholesterol in participants who lost more weight. They also had a greater reduction in HbA1c, both in those with and without diabetes. For example, participants with diabetes who lost ≥5% body weight had a median reduction in HbA1c of 5 [2, 14] mmol/mol, compared to a reduction of 2 [-1, 6] in intermediate responders and an increase in HbA1c of 1 [-4, 4] mmol/mol in those who did not lose weight (p=0.0001). Responders also had a greater reduction in their depression score (p=0.0001) and in four of nine Dartmouth COOP domains, as shown in **Table 5**.

**Table 5:** Changes in metabolic, cardiovascular, psychological and quality-of-life outcomes in CLANN programme completers, by category of weight loss.

Variable Name	All Participants	Non-Responders (No weight loss/weight gain)	Intermediate Responders (0.1-4.99% Weight loss)	Responders (≥5% weight loss)	p-value
N	815	257	454	104	
Percentage Weight Change (%)	-1.3 [-3.3, 0.4]	1.4 [2.5, 0.5]	-1.7 [-3.1, -0.9]	-6.9 [-9, -5.7]	**0.0001**
Weight Change (Kg)	-1.6 [-4.4, 0.5]	1.8 [0.7, 3.2]	-2.2 [-4, -1.2]	-9.5 [-12.2, -7.4]	**0.0001**
Δ Waist (cm)	-3 [-6.5, 0]	0 [-3, 3]	-3.3 [-7.4, -0.5]	-7 [-10, -3]	**0.0001**
Δ Systolic BP (mmHg)	-16 [-26, -5]	-16 [-27, -5]	-17 [-26, -5]	-16 [-31, -5]	0.59
Δ Diastolic BP (mmHg)	-1 [-7, 4]	0 [-6, 6]	-1 [-7, 4]	-3 [-10, 4]	**0.029**
Δ Maximum METs	0.7 [0, 1.95]	0.7 [0, 1.4]	0.7 [0, 2.1]	1 [0.3, 3.8]	**0.0025**
Metabolic:					
Δ Total Cholesterol (mmol/l)	-0.1 [-0.5, 0.2]	0 [-0.4, 0.2]	-0.1 [-0.4, 0.2]	-0.3 [-0.6, 0]	**0.0004**
Δ LDL Cholesterol (mmol/l)	-0.1 [-0.4, 0.2]	0 [-0.4, 0.3]	0 [-0.3, 0.2]	-0.2 [-0.5, 0.1]	**0.017**
Δ HDL Cholesterol (mmol/l)	0 ± 0.2	0 ± 0.2	0 ± 0.2	-0.1 ± 0.2	**0.0043**
Δ Triglycerides (mmol/l)	0 [-0.3, 0.2]	-0.1 [-0.3, 0.3]	0 [-0.3, 0.2]	-0.1 [-0.3, 0.1]	0.45
Δ HbA1c (mmol/mol) (No DM)	0 [-2, 1]	0 [-1, 1]	0 [-2, 1]	-2 [-4, 0]	**0.0001**
Δ HbA1c (mmol/mol) (DM)	-2 [-6, 1.6]	1 [-4, 4]	-2 [-6, 1]	-5 [-14, -2]	**0.0001**
HADS Scores:					
Δ Depression Score	-2 [-4, 0]	-1 [-3, 0]	-2 [-5, 0]	-3 [-5, -1]	**0.0001**
Δ Anxiety Score	-1.4 ± 3.3	-1.1 ± 3.4	-1.5 ± 3.4	-1.8 ± 3	0.2
**Δ EQVAS Score**	10.7 ± 20.6	9.9 ± 20.3	10.3 ± 20.7	14 ± 20.8	0.28
Dartmouth COOP:					
Δ Dartmouth Physical	-0.8 ± 1.1	-0.7 ± 1.1	-0.7 ± 1.1	-0.9 ± 0.9	0.63
Δ Dartmouth Feelings	-0.4 ± 1.2	-0.2 ± 1.2	-0.4 ± 1.1	-0.7 ± 1	**0.0018**
Δ Dartmouth Daily Activity	-0.4 ± 1.1	-0.4 ± 1.1	-0.4 ± 1.1	-0.6 ± 1.1	0.39
Δ Dartmouth Social Activity	0 {-1, 0]	0 {-1, 0]	0 {-1, 0]	0 {-2, 0]	0.035*
Δ Dartmouth Pain	-0.2 ± 1.2	-0.1 ± 1.2	-0.2 ± 1.2	-0.3 ± 1.2	0.27
Δ Dartmouth Change	-0.6 ± 1.1	-0.5 ± 1	-0.6 ± 1	-0.9 ± 1.1	**0.0011**
Δ Dartmouth Overall Health	-0.6 ± 1	-0.5 ± 0.9	-0.6 ± 1	-0.6 ± 1	0.63
Δ Dartmouth Support	-0.2 ± 1.3	-0.2 ± 1.2	-0.2 ± 1.3	-0.2 ± 1.5	0.88
Δ Dartmouth Quality of Life	-0.3 ± 0.9	-0.2 ± 0.9	-0.3 ± 0.8	-0.5 ± 0.9	**0.012**

“Non-responders” were defined as having no weight loss or some weight gain, “intermediate responders” lost between 0.1 and 4.99% and “responders” were defined as those who lost 5% or more of their total body weight.

Normally distributed changes in variables are presented as means ± standard deviations, while non-normally distributed changes in variables are presented as medians and [interquartile range]. Comparisons across weight loss categories for normally distributed variables are made with one-way ANOVA (and Bonferroni-adjusted pairwise comparisons) and for non-normally distributed variables, the Kruskal Wallis test (and Dunn pairwise test for pairwise comparisons).

*Denotes p value for pairwise comparisons between non-responders and responders.

DM: (Type 2) Diabetes Mellitus. P values <0.05 have been highlighted in bold.

Finally, as shown in **Table 6**, in unadjusted linear regression analyses where weight loss percentage was treated as a continuous independent/exposure variable, it was associated with statistically significant improvements in waist circumference, systolic and diastolic blood pressure, fitness, all components of the lipid profile (except triglycerides), HbA1c, depression, anxiety and quality of life scores, as well as improvements in six of nine Dartmouth COOP domains. Most of these associations remained after adjusting for confounders, except that the association between weight loss and reductions in blood pressure, the “daily activity” domain of the Dartmouth COOP questionnaire, HDL- and LDL-cholesterol were not statistically significant after adjusting for baseline BMI and MET_max_.

**Table 6:** Associations between percentage weight loss and changes in metabolic, cardiovascular, psychological and quality-of-life outcomes in CLANN programme completers.

	β	95% Confidence Interval	p-value	β	95% Confidence Interval	p-value	β	95% Confidence Interval	p-value
	Unadjusted:			Adjusted Age and Sex:			Adjusted Age, Sex, Attendance, Baseline BMI and Fitness		
Anthropometric:									
Δ Waist (cm)	-0.78	[-0.901, -0.659]	**<0.0001**	-0.805	[-0.926, -0.683]	**<0.0001**	-0.811	[-0.936, -0.685]	**<0.0001**
Δ Systolic BP (mmHg)	-0.421	[-0.776, -0.065]	**0.021**	-0.345	[-0.699, 0.008]	0.055	-0.349	[-0.721, 0.024]	0.066
Δ Diastolic BP (mmHg)	-0.313	[-0.505, -0.121]	**0.001**	-0.303	[-0.497, -0.108]	**0.002**	-0.205	[-0.423, 0.012]	0.064
Δ Fitness (METs)	0.089	[0.043, 0.134]	**<0.0001**	0.091	[0.046, 0.137]	**<0.0001**	0.094	[0.051, 0.136]	**<0.0001**
Metabolic:									
Δ Total Cholesterol (mmol/l)	-0.022	[-0.035, -0.008]	**0.002**	-0.022	[-0.035, -0.008]	**0.002**	-0.024	[-0.038, -0.009]	**0.002**
Δ LDL Cholesterol (mmol/l)	-0.014	[-0.025, -0.002]	**0.02**	-0.013	[-0.025, -0.002]	**0.024**	-0.013	[-0.026, 0.001]	0.06
Δ HDL Cholesterol (mmol/l)	-0.004	[-0.008, -0.0004]	**0.03**	-0.004	[-0.008, -0.0002]	**0.035**	-0.003	[-0.007, 0.002]	0.25
Δ Triglycerides (mmol/l)	-0.015	[-0.036, -0.007]	0.18	-0.013	[-0.034, -0.008]	0.24	-0.018	[-0.04, -0.003]	0.091
Δ HbA1c (mmol/mol) (No DM)	-0.258	[-0.35, -0.166]	**<0.0001**	-0.248	[-0.342, -0.154]	**<0.0001**	-0.219	[-0.304, -0.134]	**<0.0001**
Δ HbA1c (mmol/mol) (DM)	-0.78	[-1.128, -0.431]	**<0.0001**	-0.799	[-1.15, -0.447]	**<0.0001**	-0.955	[-1.386, -0.524]	**<0.0001**
HADS Scores:									
Δ Depression Score	-0.163	[-0.241, -0.085]	**<0.0001**	-0.175	[-0.254, -0.095]	**<0.0001**	-0.143	[-0.23, -0.056]	**0.001**
Δ Anxiety Score	-0.072	[-0.144, 0.001]	0.053	-0.076	[-0.149, -0.002]	**0.044**	-0.065	[-0.146, 0.017]	0.12
Δ EQVAS Score:	0.555	[0.087, 1.023]	**0.02**	0.659	[0.186, 1.132]	**0.006**	0.64	[0.107, 1.174]	**0.019**
Dartmouth COOP:									
Δ Dartmouth Physical	-0.014	[-0.037, 0.01]	0.26	-0.013	[-0.037, 0.011]	0.28	-0.015	[-0.043, 0.013]	0.3
Δ Dartmouth Feelings	-0.04	[-0.064, -0.015]	**0.002**	-0.042	[-0.067, -0.016]	**0.001**	-0.04	[-0.068, -0.012]	**0.006**
Δ Dartmouth Daily Activity	-0.025	[-0.048, -0.001]	**0.038**	-0.024	[-0.048, -0.0001]	**0.049**	-0.021	[-0.047, 0.006]	0.13
Δ Dartmouth Social Activity	-0.036	[-0.064, -0.009]	**0.01**	-0.038	[-0.066, -0.0098]	**0.008**	-0.042	[-0.074, -0.011]	**0.009**
Δ Dartmouth Pain	-0.017	[-0.043, 0.009]	0.19	-0.02	[-0.046, 0.006]	0.13	-0.025	[-0.054, 0.004]	0.095
Δ Dartmouth Change	-0.052	[-0.075, -0.03]	**<0.0001**	-0.051	[-0.074, -0.028]	**<0.0001**	-0.045	[-0.07, -0.02]	**<0.0001**
Δ Dartmouth Overall Health	-0.02	[-0.041, 0.0003]	**0.054**	-0.022	[-0.043, 0.001]	**0.038**	-0.027	[-0.05, -0.004]	**0.021**
Δ Dartmouth Support	-0.006	[-0.034, 0.023]	0.71	-0.001	[-0.03, 0.028]	0.95	-0.001	[-0.032, 0.032]	0.98
Δ Dartmouth Quality of Life	-0.032	[-0.051, -0.013]	**0.001**	-0.035	[-0.053, -0.016]	**0.001**	-0.033	[-0.054, -0.011]	**0.003**

Associations between percentage weight loss and other variables are expressed as β- coefficients and [95% confidence intervals]. P values <0.05 have been highlighted in bold.

## Discussion

Our findings have yielded a number of important insights into the factors associated with the magnitude of health gains from a structured lifestyle modification programme for adults with severe obesity. Firstly, older patients, those with lower measures of depression and anxiety and those with better perceived quality of life were more likely to complete the programme rather than to drop out, but sex, educational attainment, employment status or marital status had no effect. Secondly, the presence of comorbidities (other than depression) like diabetes, cardiovascular disease or smoking had no influence on drop-out rates. Thirdly, we were surprised that “access” to the programme, in terms of distance travelled to the facility where it was delivered did not influence drop-out rates, though longer waiting time to start the programme was associated with a lower completion rate.

Similar findings emerged when we examined the influence of these factors on weight loss in patients who completed the programme. The 12.8% of programme completers who were “responders” (losing 5% or more of their body weight) were more likely to be male, consistent with observations in a similarly-sized cohort of bariatric patients in the UK who underwent a liver reduction diet prior to bariatric surgery (17). Older age was also associated with a lower chance of dropping out of the programme, and a higher chance of achieving at least 5% weight loss. This is consistent with observations in trials of structured lifestyle interventions in primary care in adults with overweight or obesity (16), in those undergoing dietary intervention prior to bariatric surgery (17) and for the prevention of diabetes in people with impaired glucose metabolism (31). Future studies will need to explore the implications of these findings for clinical practice, but they suggest that upper age limits for inclusion in structured lifestyle programmes are unwarranted.

Those with lower anxiety scores or, paradoxically, commuting further to the intervention were more likely to be responders, while retired participants were much less likely to be responders. As anticipated, weight loss was proportional to the number of programme sessions attended. Despite our relatively crude quantification of physical activity (using a questionnaire to determine whether 150 minutes per week of at least moderate intensity activity was achieved), it is noteworthy that weight loss responders had a higher proportion of patients achieving this threshold at follow-up, compared to non-responders. This is consistent with findings in patients with obesity (32), bariatric surgery patients (33) and in the Look AHEAD trial of structured lifestyle modification to treat type 2 diabetes (34, 35), where increased physical activity was shown to be associated with greater longer term weight loss. Our findings emphasise the importance of combining dietary and physical activity components in structured lifestyle programmes, in line with current guidance (36, 37).

Only 12.8% of our programme completers lost 5% or more of their body weight. We think that this apparently modest response is due to the short duration of the programme, rather than a lack of effectiveness of the intervention – something it is not possible to assess formally in a prospective cohort study such as this. Nonetheless, the strong and consistent associations we observed between the amount of weight lost and improvements in anthropometric, metabolic, cardiovascular, mental health and quality of life outcomes are noteworthy for several reasons. Irish studies in patients with severe obesity have been very limited to date. While the negative impact of severe obesity on self-reported quality of life is well established (38, 39), ours is the first study to describe the associations between the magnitude of purposeful weight loss in a structured lifestyle modification programme and changes in the EQVAS and five of nine Dartmouth COOP domains in Irish adults with severe obesity. There is a high burden of depression (40) and anxiety (41) in people with obesity, and purposeful weight loss with lifestyle modification has previously been shown to reduce anxiety and depression scores (42, 43) in some studies, but not all: Of note, in Look-AHEAD, anxiety and depression scores deteriorated in both lifestyle intervention groups, with no difference in antidepressant medication after 10 years of follow-up (44). Other trials have shown improvements in mental health outcomes in participants randomised to weight loss interventions (45). Moreover, the health benefits of lifestyle interventions in patients with severe mental illness who have overweight or obesity are well described (46, 47). However, ours is the first study to show that the amount of weight lost with structured lifestyle modification is proportional to the improvements in mental health obtained in patients with severe obesity.

It is important to note that the improvements in blood pressure and lipid profiles with weight loss occurred while maintaining baseline medication usage throughout the intervention and did not occur as a result of intensification of antihypertensive or lipid lowering therapy. Similarly, improvements in HbA1c with weight loss in patients with type 2 diabetes occurred independently of medications, which were not changed. The observation of an influence of weight loss on HbA1c, even in participants without diabetes and after just ten weeks of follow-up, suggests important metabolic benefits from purposeful weight loss that ought to be explored in future studies and which are consistent with our previous observations of improvements in insulin sensitivity after bariatric surgery (48) and intensive meal replacement programmes (49) in this patient group.

Our study has some limitations, such as the relatively short duration of follow-up. Whether these benefits are sustained in the longer term remains to be seen. We have limited information about the reasons patients did not start the programme (unspecified in 26.2%) or did not complete the programme (unspecified in 44.9%). Without a control group and a randomised controlled trial design, we cannot make inferences about the efficacy and effectiveness of the intervention, compared to not receiving the intervention. Participation and completion rates were relatively high, but our findings may not be generalisable to all patients with severe and complicated obesity. For example, people who are seeking medical help for treatment of their severe and complicated obesity, and are willing to attend hospital services, may respond inherently differently to structured lifestyle intervention compared to those who have not sought clinical assistance. The generalisability of our findings is also limited by the geographical location of the intervention in the West of Ireland – whether similar associations between weight loss with structured lifestyle modification and mental health gains exist in other jurisdictions, climates and healthcare systems remains to be determined.

It is important to note that for the two sets of regression analyses presented in this paper – one exploring the potential predictors of whether or not participants completed the programme, and the second examining the potential predictors of successful attainment of ≥5% weight loss in those participants who completed the programme, we adopted an inclusive approach, using any clinically plausible variable that was available in our dataset. This represents an additional limitation of our study, in that variables were included if they were available and deemed by us to be plausible contributors to chances of completion or successful weight loss. We think that it is likely that the same variables that might give rise to a lower likelihood of completion (such as a diagnosis of depression) or waiting time to start the intervention might also adversely affect the clinical response to the programme, which is why the same potential predictor variables were included for treatment and response. The variables were chosen on the basis of availability and likely relevance. We think that age, sex, smoking status, wait time to start intervention, distance from intervention, marital and employment status and eligibility for state sponsored medical care are relevant and highly plausible variables for consideration. We think that the consideration of self-reported physical activity at baseline was important, as other studies have found it to be associated with better outcomes after bariatric surgery (33) and structured lifestyle modification to treat type 2 diabetes (34, 35).

A further limitation is that although anthropometric measurements were carried out by trained staff, they were not blinded to participant treatment status and these staff were also involved in intervention delivery. This may have introduced bias to measures of waist circumference or fitness for example, however unintentionally. A randomised controlled trial with assessor blinding would overcome this limitation, which should be borne in mind with future studies. Another limitation is that for the purpose of these analyses we have taken a very narrow view of treatment success, using an arbitrarily defined weight-loss threshold (28), and it is important to recognise the potential benefits of these programmes even for patients where substantial weight loss does not occur.

Our study also has a number of strengths. To our knowledge it represents the largest single-centre cohort study of bariatric patients undergoing such an intervention, not just in Ireland but globally, where important fitness, mental health and quality of life outcomes have been assessed. The consistency of the duration and method of intervention delivery has minimised the effect of variations in intervention exposure on response heterogeneity, insofar as possible.

## Conclusions

In adults with severe obesity completing a ten-week structured lifestyle modification programme, older age and good mental health were predictors of programme completion and the successful attainment of ≥5% weight loss. Improvements in cardiovascular and metabolic outcomes were proportional to the amount of weight lost. Our finding that improvements in mental health were also proportional to the amount of weight lost is particularly novel. Overall, these are important new observations and insights regarding the factors influencing the response to structured lifestyle modification in patients with severe obesity, and highlight the major health benefits of purposeful weight loss, where it is achieved.

## Data availability statement

The raw data supporting the conclusions of this article will be made available by the authors, without undue reservation.

## Ethics statement

The studies involving humans were approved by Galway University Hospitals Research Ethics Committee (reference CA 1070). The studies were conducted in accordance with the local legislation and institutional requirements. The participants provided their written informed consent to participate in this study.

## Author contributions

FF: Conceptualization, Data curation, Formal Analysis, Funding acquisition, Investigation, Methodology, Project administration, Resources, Writing – original draft, Writing – review & editing. IG: Conceptualization, Project administration, Resources, Writing – review & editing. RH: Data curation, Formal Analysis, Funding acquisition, Investigation, Writing – review & editing. EM: Formal Analysis, Investigation, Methodology, Writing – review & editing. LH: Data curation, Investigation, Project administration, Resources, Supervision, Writing – review & editing. AH: Data curation, Investigation, Project administration, Resources, Supervision, Writing – review & editing. BM: Formal Analysis, Methodology, Resources, Writing – review & editing. MH: Conceptualization, Methodology, Resources, Writing – review & editing. CC: Resources, Writing – review & editing. KC: Conceptualization, Methodology, Resources, Writing – review & editing. SS: Project administration, Resources, Writing – review & editing. JJ: Conceptualization, Data curation, Funding acquisition, Investigation, Methodology, Project administration, Resources, Supervision, Writing – review & editing. TO: Resources, Supervision, Writing – review & editing. MO: Conceptualization, Formal Analysis, Investigation, Methodology, Resources, Supervision, Writing – original draft, Writing – review & editing.
