# Supplementary material for: Factors associated with weight loss and health gains in a structured lifestyle modification programme for adults with severe obesity: a prospective cohort study
Source: Front Endocrinol (Lausanne). 2023 Oct 17;14:1257061. doi: 10.3389/fendo.2023.1257061 (PMC10616877; doi:10.3389/fendo.2023.1257061)
Supplement: Supplementary file 1 [file Table_1.docx]

**Supplementary Table 1:** Baseline Demographic Characteristics of Patients with Severe Obesity who Completed or Did Not Complete the CLANN Structured Lifestyle Modification Programme.

| **Variable Name** | **Completers** | | | **Non-Completers** | | | **p-value** |
| --- | --- | --- | --- | --- | --- | --- | --- |
|  |  |  |  |  |  |  |  |
| n | 877 |  |  | 245 |  |  |  |
| Distance to Centre (km) | 65 | [25, | 100] | 60 | [20, | 100] | 0.89 |
| Lives >40 km from centre | 577 | (65.9) |  | 157 | (64.1) |  | 0.59 |
| Wait time (days) | 73 | [27.5, | 171.5] | 85 | [31, | 184] | 0.13 |
| Current drinker | 244 | (38.9) |  | 58 | (32) |  | 0.092 |
| **Employment Status:** |  |  |  |  |  |  | 0.098 |
| Employed | 300 | (35.5) |  | 71 | (31.8) |  |  |
| Self-employed | 53 | (6.3) |  | 16 | (7.2) |  |  |
| Unemployed | 107 | (12.7) |  | 25 | (11.2) |  |  |
| Retired | 100 | (11.8) |  | 18 | (8.1) |  |  |
| Family Carer | 155 | (18.3) |  | 42 | (18.8) |  |  |
| Student/ training scheme | 41 | (4.9) |  | 12 | (5.4) |  |  |
| Disability Benefit | 89 | (10.5) |  | 39 | (17.5) |  |  |
| **Education Level:** |  |  |  |  |  |  | 0.76 |
| Primary or less | 209 | (27.4) |  | 50 | (26) |  |  |
| Secondary level | 263 | (34.4) |  | 63 | (32.8) |  |  |
| Third level or higher | 292 | (38.2) |  | 79 | (41.2) |  |  |
| **Lives with partner?:** |  |  |  |  |  |  | 0.62 |
| Yes | 527 | (61.4) |  | 145 | (59.7) |  |  |
| No | 331 | (38.6) |  | 98 | (40.3) |  |  |
| **Marital status:** |  |  |  |  |  |  | 0.86 |
| Married | 465 | (54) |  | 123 | (51.9) |  |  |
| Single | 258 | (30) |  | 71 | (30) |  |  |
| Divorced/Separated | 67 | (7.8) |  | 20 | (8.4) |  |  |
| Permanent Partnership | 50 | (5.8) |  | 18 | (7.6) |  |  |
| Widowed | 21 | (2.4) |  | 5 | (2.1) |  |  |
| **GMS eligible?:** |  |  |  |  |  |  | **0.019** |
| Yes | 545 | (67.6) |  | 169 | (75.8) |  |  |
| No | 261 | (32.4) |  | 54 | (24.2) |  |  |

None of the continuous variables in the table were normally distributed, so all are presented as medians and [inter-quartile range]. Proportions are expressed as the number of participants and (percentage). Comparisons of baseline measures of continuous variables between programme completers and non-completers were made using the Wilcoxon Rank Sum (Mann-Whitney U) Test. Comparisons of proportions between the two groups for categorical variabels were made using the Chi-Square test.

GMS: General Medical Services, refers to the means-tested provision of state-sponsored care.
